# Higuchi Dimension of Digital Images

**DOI:** 10.1371/journal.pone.0024796

**Published:** 2011-09-13

**Authors:** Helmut Ahammer

**Affiliations:** Institute of Biophysics, Centre of Physiological Medicine, Medical University of Graz, Graz, Austria; University of Milano-Bicocca, Italy

## Abstract

There exist several methods for calculating the fractal dimension of objects represented as 2D digital images. For example, Box counting, Minkowski dilation or Fourier analysis can be employed. However, there appear to be some limitations. It is not possible to calculate only the fractal dimension of an irregular region of interest in an image or to perform the calculations in a particular direction along a line on an arbitrary angle through the image. The calculations must be made for the whole image. In this paper, a new method to overcome these limitations is proposed. 2D images are appropriately prepared in order to apply 1D signal analyses, originally developed to investigate nonlinear time series. The Higuchi dimension of these 1D signals is calculated using Higuchi's algorithm, and it is shown that both regions of interests and directional dependencies can be evaluated independently of the whole picture. A thorough validation of the proposed technique and a comparison of the new method to the Fourier dimension, a common two dimensional method for digital images, are given. The main result is that Higuchi's algorithm allows a direction dependent as well as direction independent analysis. Actual values for the fractal dimensions are reliable and an effective treatment of regions of interests is possible. Moreover, the proposed method is not restricted to Higuchi's algorithm, as any 1D method of analysis, can be applied.

## Introduction

Digital images are increasingly utilized to represent data in all kinds of sciences. They can be used for visual or graphical purposes only or for a closer investigation of an object via image processing techniques. If the objects in an image are not geometrically regular—which is often the case for natural objects such as landscapes, animals or cells—both the interpretation and the classification can be important. For these tasks, determining the fractal dimensions of 2D digital images has been very successful in recent years [1]–[5]. The methods involved include the well known Box counting method or the Minkowski dilation method [3]. It is also possible to use gray value statistics [6], differential box counting [7], a variation method [8], a blanket method [9] or frequency analysis [10]–[12]. Despite the effectiveness of these methods, they have some serious limitations. Very often the object of interest does not fill the digital image entirely, but instead is surrounded by a background, e.g., a light microscopic image of a single cell surrounded by culture medium, an electron microscopic image of a cell nucleus surrounded by stroma or a histological image of a special tissue surrounded by neighbouring tissue. In all these cases, it would be necessary to calculate the properties or fractal dimensions only for the regions of interest, without incorporating any information from the background. Furthermore, it is not possible to calculate the fractal dimension of a specific line or curve through an image. Such a line or curve can be considered to be nothing more than a long region of interest without a width or with a width of one pixel.

The present work proposes a new method to overcome these limitations by using 1D signal analysis methods. 2D images are either projected onto 1D signals or several image rows, columns, radial lines or spirals are extracted in order to gather a batch of 1D signals. Projection leads to a loss of information, but has the advantage of drastically decreased computational requirements. Extraction of rows and/or columns does not imply a loss of information, and the fractal dimension of the whole image can be calculated very precisely.

Theoretically, an extracted 1D signal of an image is an intersection of the gray value surface with a two dimensional plane and therefore, the intersection theorem for fractals [13] can be applied:
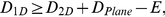
(1)with 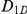 the fractal dimension of the 1D signal, 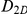 the fractal dimension of the gray value surface in a three dimensional Euclidian space 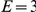, and a plane with 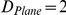. Usually the greater than relation can be replaced by equality. Then, the fractal dimension range 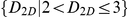 of the surface yields an expected fractal dimension range of 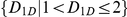 for the 1D signal or profile. Projection in this context is a data reduction by summing up the grey values along an axis. For this sort of projection the projection slice theorem is valid, which is commonly applied for inverse problems, such as computed tomography. A single projection integrates the original data, unavoidably yielding a loss of high frequency components. Nevertheless, it is feasible to calculate quantitative parameters describing the data set, e.g. the fractal dimension. It turned out that projection yields in many cases quite similar, mainly a little lower values compared to extraction methods, but, in some cases, it can lead to false values, which is described and elaborated thoroughly in the result and discussion sections.

One dimensional data is commonly a time series of data points, which can be examined by a very wide range of excellent linear as well as nonlinear methods. While there exist a huge range of methods concerning 1D signal processing and signal analyses (e.g. 1D filtering algorithms), this study is focused on nonlinear methods studying fractal dimensions of objects. These 1D nonlinear analyses are mainly performed in the investigation of nonlinear dynamical systems [14]–[16], bifurcations [17] or even critical transitions [18]. The range of possible methods includes phase space analysis, attractor analysis, Fourier methods, the Higuchi method [19] and others.

Despite of the effectiveness of these 1D methods, there have been only very limited efforts to expand these methods to 2D in the past. There are a few exceptions [20]–[22], but because of their rarity, there is a very high potential for improving and expanding the classical 2D methods. This work intends to pursue these promising approaches. The proposed methods include some generally applicable techniques, which can be adapted very easily to actual problems.

## Methods

### Digital images

**Figure 1:**
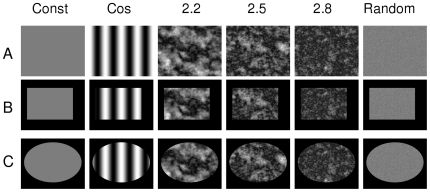
Six sample images. **A** An image with constant gray value, an image with cosine shaped varying gray levels in the horizontal direction and constant gray value in the vertical direction, three images with varying gray levels but distinct predefined fractal dimensions and finally an image with random gray values. **B** Same six images as in A, but with a rectangular region of interest (ROI). **C** Same six images as in A, but with an elliptical ROI.

Several digital gray level images were generated in order to test the calculations of the images' Higuchi dimensions. The varying gray level surface of a 2D image can be interpreted as a 3D landscape in a three dimensional embedding space. The following images were constructed (Figure 1A): An image with constant gray value, an image with a cosine shaped variance of gray levels in the horizontal direction and a constant gray value in vertical direction, three images with varying gray levels but predefined fractal dimensions, and finally an image with random gray values.

The fractal gray level landscapes were constructed using an inverse Fourier method described in [23]. Briefly, an artificial, randomly distributed Fourier power spectrum is constructed. The value of the desired fractal dimension, 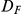, is taken to calculate the slope 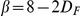.Then, *β* is used to create a corresponding power spectrum. Applying the inverse Fourier transformation with arbitrary phase values gives a gray value surface with the desired fractal dimension 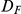.

Two artificial regions of interest (ROI) were constructed, one rectangular and one elliptical, by setting all pixel values outside of the ROI to zero. These images can be seen in Figure 1B&C. The actual shapes of the ROIs were chosen with unsymmetrical distances to the image border in order to simulate an actual case.

All images had an identical resolution of 1300×1030 pixels, which is high enough for the calculations intended [24] and resemble a commonly used image size. The images were saved as 8 bit gray level images in tiff format.

The images were constructed with IDL (Interactive Data Language, ITT Industries Inc., Boulder, USA).

### Construction of 1D data sequences

There is not a standard procedure for constructing 1D data point series out of 2D digital images. At first glance, a reduction of order seems to inevitably cause a loss of information. But this loss does not always occur without exception. The amount of lost information is strongly dependent on the actual reduction process. In practice, there exist a huge number of possibilities to extract 1D signals out of 2D images. Extractions of rows or columns, along radial lines, spirals or arbitrary curves or stitching together rows or columns, to name but a few, are possible. In fact, the proposed method of calculating fractal dimensions is not restricted to any special type of extraction and therefore, exemplarily the following extraction algorithms were chosen for this study:

- The gray values are projected vertically to the x-axis and horizontally to the y-axis. This projection resembles the summing up of gray values, and two 1D signals are constructed.
- Every horizontal row and every vertical column of the image is extracted and taken as a separate 1D signal. This approach leads to (*n*+*m*)-many signals, with *n* the number of image columns and *m* the number of image rows.
- Radial lines through the centre of the image with a subsequent angle difference of 1° are extracted. Therefore, 180 signals cover the range from 0 to 2π.
- An Archimedean spiral starting at the centre of the image and turning 10 times through the image is extracted.

The evaluation time is considerably low for method (i) and only marginally higher for (iv). The time for (ii) is (*n*+*m*)/2 times and for (iii) 90 times higher than the time for (i). On a standard PC (for the images with a resolution of 1300×1030 pixels), the calculations (including the display of graphical user interfaces and the display of every single regression plot) using method (i) took <0.15 minutes, whereas for method (ii) they took about 200 minutes and for method (iii) about 15 minutes per image. Parallelization of the algorithms, especially for method (ii) and (iii) would be possible, because the individual 1D signals can be independently processed.

The results of the individual signals can also be grouped together by calculating mean values. Therefore, it is possible to get distinct mean values for the x- and/or y-direction or one single value for the whole image.

All the images were additionally investigated and examined with two different ROIs: a rectangular and an elliptical shape. Outside of the ROI, the gray values were set to zero, so each of the 1D signals showed both leading and tailing zeros. Zero gray values were interpreted as being the background. Obviously, the fractal dimension calculations strongly depended on these leading and tailing zeros, and it was not possible to neglect this influence. In order to examine this influence, the calculations were carried out in two ways. First, the calculations were straightforwardly carried out by including the zeros (inclusive background), and second, the calculations were carried out after both the leading and the tailing zeroes were excluded (exclusive background).

### Higuchi dimension

The Higuchi dimension, 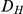, is a measure of irregularity and is calculated for time series directly in the time domain [19]. The calculations are carried out without phase space constructions. Several lengths, 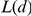, of the signal or curve are calculated, and a double logarithmic plot, 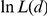
versus 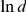, is used to estimate the actual dimension value. The assumption is that a fractal signal scales according to the following:
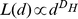
(2)The discrete data point series 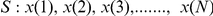, with *N* the total number of data points, must consist of values or observations at regular intervals. From this single data point series, *d* new data point series 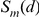, with 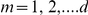, where *m* is the initial time and *d* a time interval, are constructed.
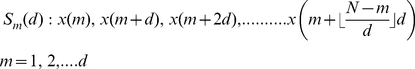
(3)For each 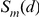, the lengths 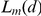 are calculated as follows.
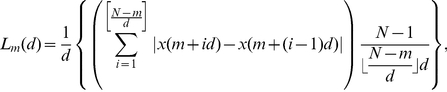
(4)where *m* and *d* are integers and 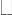 denotes the floor function. The lengths 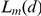 are the normalized sums of the differences of the values, with a distance of *d* and a starting point *m*. For each *d*, the mean 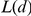 is calculated as follows.
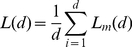
(5)Finally, the slope of a linear regression of a double logarithmic plot of 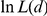 and 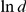 gives the Higuchi dimension, 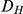. The maximal interval 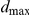 was determined by plotting several regressions with subsequently increasing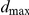. For each individual regression, the coefficient of determination 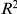 was calculated. The saturation point, where 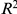 did not increase significantly was taken for the maximal *d*. Actually, 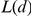 was calculated for 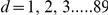, and the best linear regression (again by checking 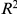) in the double logarithmic plot was gained for the range 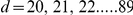. This range of *d* resulted in the best estimations of the theoretical dimension values.

The values of the Higuchi dimension, 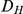, of a 1D curve *S* always fall in the closed interval [1], [2]. There is one exception, when all the data point values have a constant value. In that case, all the differences in the summation of 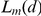 are all zero, resulting in 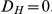 A simple curve, such as a sine or cosine function, has a dimension 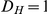. The other extreme is a randomly distributed curve with 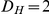. The dimension for fractals lies between 1 and 2.

### Fourier dimension

Frequency analysis, and in particular the FFT (Fast Fourier Transformation), is widely applied in image processing, and the fractal dimension 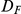, also called the “Fourier dimension,” is related to the power spectrum of a 2D image. The power spectrum is given by:
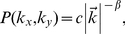
(6)with 
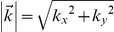
, and c is a constant.


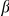
 can be examined by fitting the function in Equation (6) to the calculated two dimensional power spectrum. By taking the logarithm, the least squares approximation gives:
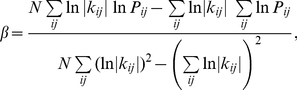
(7)with 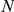 the number of data points and *i* and *j* the indices in the horizontal and vertical directions respectively.

The fractal dimension, 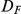, of 2D images, having a topological dimension 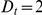, can be estimated with the following equation:
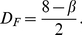
(8)The range of possible values is between 2 and 3.

The calculations were carried out with IDL (Interactive Data Language, ITT Industries Inc., Boulder, USA).

Both dimensions, the Fourier dimension as well as the Higuchi dimension depend on the construction of a power law of distinct quantities. Although these quantities are not identical, the power law reflects the intrinsic nonlinear relation of these distinct quantities. Therefore, the slopes of the linear fits give estimates rather than exact values for the fractal dimension.

## Results

The dimension values of distinct images were examined according to each of the individual methods. Firstly, projection, extraction of rows, columns, radial lines or spirals was carried out to get 1D signals for the calculation of the Higuchi dimension. For comparison, the images were used to calculate the Fourier dimension. The slopes of the linear regressions of double logarithmic plots were determined, and the estimated values of the fractal dimensions were calculated by linear regressions.

### Linear regressions

**Figure 2:**
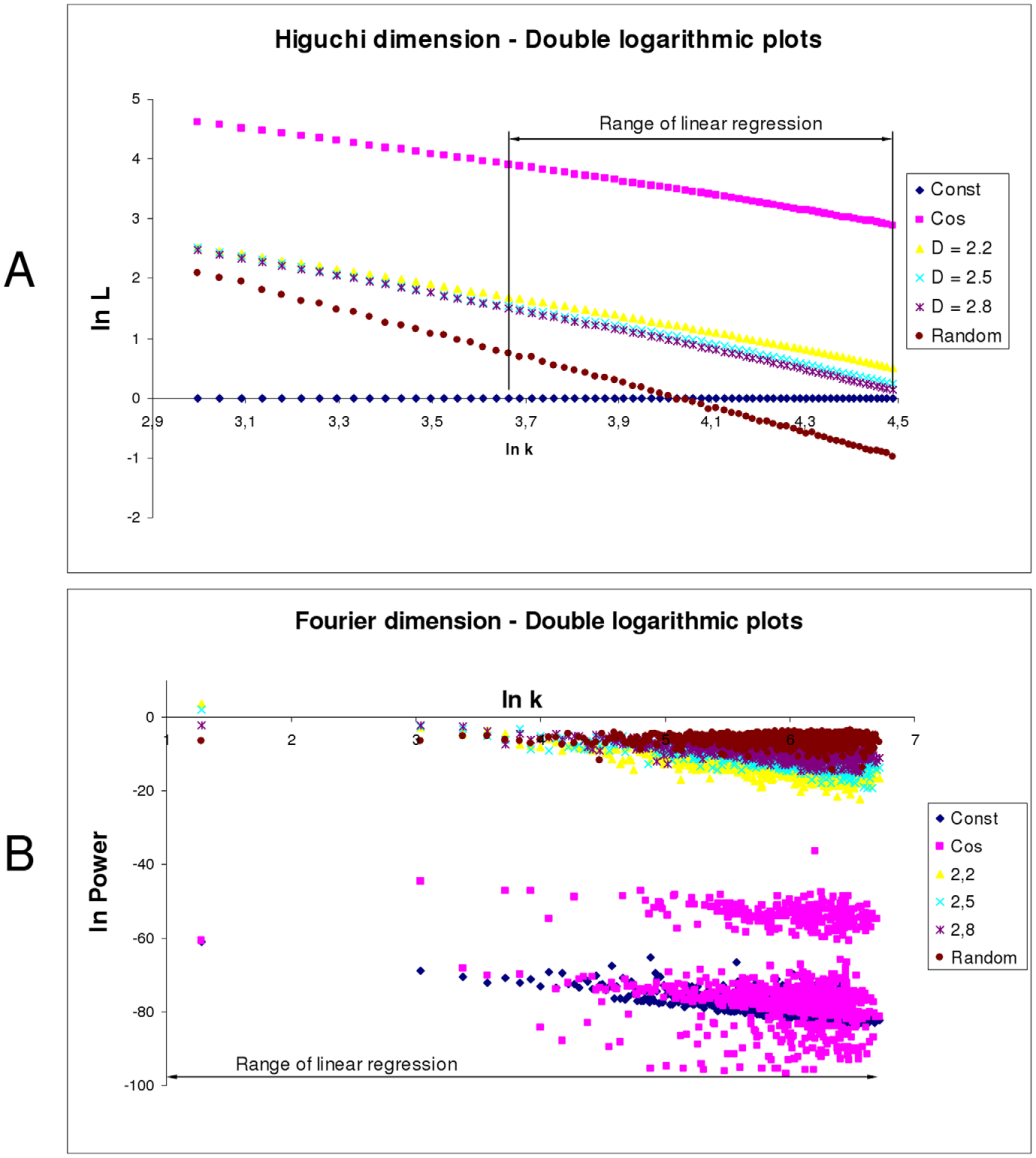
Double logarithmic plots of the Higuchi and Fourier dimension. The individual ranges of linear regressions are depicted. **A** The slopes of the Higuchi dimension show a slight tendency for two linear regions. Thus, the range of linear regression was limited to the second linear region in order to gain the best absolute dimension values. The linear regression fit the data very well, with coefficients of determination R^2^ higher than 0.993. **B** The plot data of the Fourier dimension are highly dispersed. The coefficients of determination R^2^ were about 0.332. The highest value was 0.664.

Sample double logarithmic plots and linear regressions can be seen in Figure 2. The linear regressions of the Higuchi method of the images in Figure 1A can be seen in Figure 2A. A close inspection shows a slight tendency for two linear regions, so the actual linear regression was restricted to the second region for values between 20 to 89. This restriction gave the best absolute values, e.g., a sinusoidal shape should have 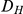 = 1, while a random shape should have 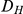 = 2. The linear regressions fit the data very well, with coefficients of determination R^2^ higher than 0.993.

The linear regressions of the Fourier method can be seen in Figure 2B. Obviously, compared to the Higuchi method, the plot data is highly dispersed, and the linear regressions did not fit the data very well. The coefficients of determination R^2^ were worse than for the Higuchi method at approximately 0.332. The highest value was only 0.664.

### Fractal dimensions of fractal shapes

As a first comparison of the Higuchi dimension analysis to the Fourier dimension analysis, gray value images, featuring a fractal surface and predefined certain fractal dimensions, were investigated. The predefined fractal dimensions were D = 2.2, D = 2.5 and D = 2.8, representing low, medium and high fractal dimensions, respectively.

**Figure 3:**
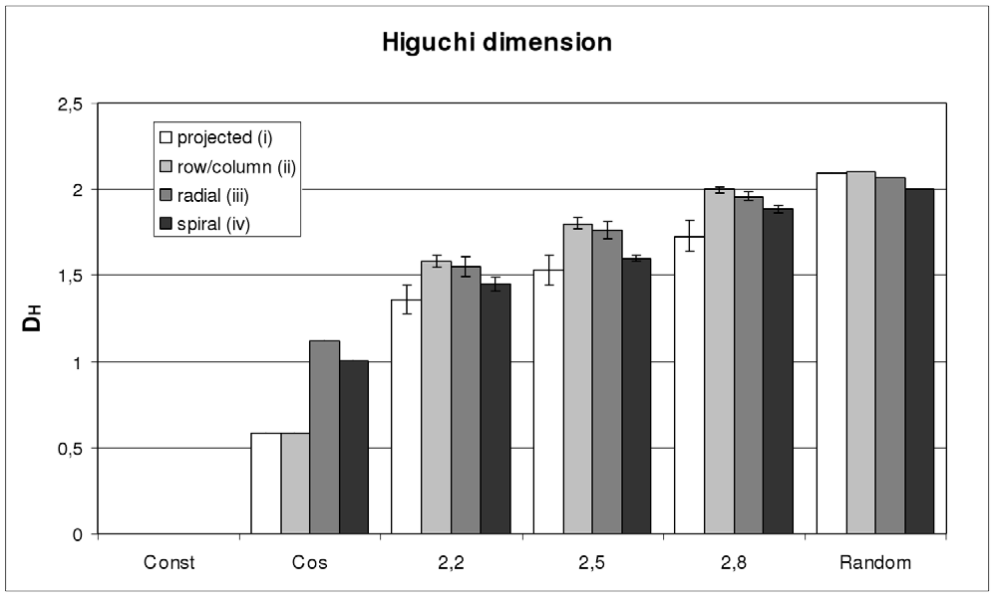
Higuchi dimensions of fractal and non-fractal images. Higuchi dimensions for an image with a constant gray value, an image with a cosine shaped gray value course in the x-direction, three images with predefined fractal dimensions (D = 2.2, 2.5, 2.8) and an image with random gray values. The legend depicts the distinct 2D to 1D methods (i)–(iv). (i) projection and averaging the values for the x- and y-direction. (ii) examining every row and column and calculations of averages. (iii) 180 radial lines through the centre of the image and calculations of averages and (iv) spirals through the image and calculations of averages.

**Figure 4:**
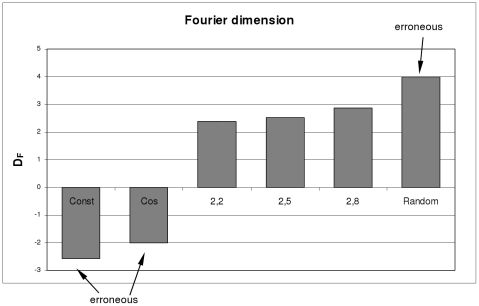
Fourier dimensions of fractal and non-fractal images. Fourier dimensions for an image with a constant gray value, an image with a cosine shaped gray value course in the x-direction, three images with predefined fractal dimensions (D = 2.2, 2.5, 2.8) and an image with random gray values. Inaccurate as well as erroneous values are emphasized with arrows.

Figure 3 and 4 show the results, and the abscissa values are the predefined fractal dimensions. For every predefined fractal dimension, 100 different images were investigated. The error bars depict the calculated standard deviations. Figure 3 shows the Higuchi dimension results for methods (i), (ii), (iii) and (iv), respectively. Figure 4 shows the results for the Fourier dimension analysis.

The values of the Higuchi dimension analysis show a continuous increase and very low levels of errors. The values for the projection method (i) were slightly smaller than for method (ii). Method (ii) and (iii) yielded quite similar values. Again method (iv) yielded marginally smaller values but not so much as method (i). As mentioned in the method section, a single value for an image was calculated.

In contrast, the values of the Fourier dimension (Figure 4) show very clearly that there are some very bad estimates. The values for fractal dimensions from 2.5 and 2.8 are estimated quite well, but the calculation for the lower value of 2.2 shows a very poor estimate.

### Fractal dimensions of non fractal shapes

The results for an image with a constant gray value, an image with a cosine shaped gray value course in the x-direction can be seen on the left side of Figures 3 and 4. The result for an image with random gray values can be seen on the right side of Figures 3 and 4.

In accordance with the theory, the constant gray value image has an estimated Higuchi dimension of zero for all four methods (i), (ii), (iii) and (iv). Furthermore, the cosine shaped varying image in the x-direction and constant values in y-direction has a Higuchi dimension of one in the x-direction and a Higuchi dimension of zero in y-direction. The average value of approximately 0.5 for methods (i) and (ii) can be seen in Figure 3. Methods (iii) and (iv) yielded a value around 1, as can be seen in Figure 3, too. Finally, the Higuchi dimension of the random image correctly shows the highest values of all.

Contrary to these positive findings for the Higuchi dimension analysis, the Fourier dimension analysis led to quite erroneous values for the non fractal images, which can be seen in Figure 4. The negative values obtained for the constant image and the cosine image are simply incorrect. The Fourier dimension 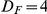 of the random dimension should instead be 3 and is therefore far too large.

### Influence of ROI

The influence of ROIs on the calculations of fractal dimensions is evident, because all the pixels outside of the ROI are zero, representing a black background. If these zeros were included in the calculations, they would definitely alter the results. Therefore, the exclusion of these pixels seems to be mandatory. Exclusion seems to be an easy way of avoiding these problems, but unfortunately this exclusion is not possible for every ROI. In fact, exclusion is only possible for a rectangular ROI, because the image inside the ROI can be extracted as a new image. For all other arbitrary shaped ROIs, there will always be some zero pixels. The influence of background effects was not examined for the Fourier dimension, due to the bad results presented so far. At this stage of development, it appears to be unnecessary to attempt to adapt the Fourier method to give reliable results, especially for ROIs. On the other hand, the Higuchi method offers great potential to overcome these ROI influences very easily. It is possible to exclude background (zeroes) prior to the dimension calculations, and the results thereby gained are shown in Tables 1 and 2 for projection method (i) and extraction method (ii), respectively. The first two rows show the “correct” values without ROIs, where an exclusion of background does not alter the results, due to an absence of zero background values.

**Table 1:** ROI influences on Higuchi Dimension using projection method (i).

ROI	Backgr.	Const	Cos	D = 2.2	D = 2.5	D = 2.8	Random
-	Incl.	0	0.59	1.36	1.53	1.73	2.09
-	Excl.	0	0.59	1.36	1.53	1.73	2.09
Rect.	Incl.	1.09^h^	1.14^h^	1.19^l^	1.32^l^	1.45^l^	1.26^l^
Rect.	Excl.	0	0.59	1.40	1.68	1.90	2.16
Ellipse	Incl.	1.29^h^	1.23^h^	1.37^l^	1.46^l^	1.52^l^	1.37^l^
Ellipse	Excl.	0	1.48^h^	1.40	1.59	1.74	2.14

h value is too low.

l value is too high.

**Table 2:** ROI influences on Higuchi Dimension using every row and column extraction method (ii).

ROI	Backgr.	Const	Cos	D = 2.2	D = 2.5	D = 2.8	Random
-	Incl.	0	0.59	1.58	1.80	1.99	2.10
-	Excl.	0	0.59	1.58	1.80	1.99	2.10
Rect.	Incl.	0.74^h^	0.75^h^	0.95^l^	1.11^l^	1.22^l^	1.28^l^
Rect.	Excl.	0	0.59	1.64	1.90	2.07	2.16
Ellipse	Incl.	1.14^h^	1.04^h^	1.23^l^	1.41^l^	1.55^l^	1.64^l^
Ellipse	Excl.	0	0.56	1.51	1.76	1.97	2.10

h value is too low.

l value is too high.

The rectangular ROI caused following distortions in case of including background (third row in the tables) compared to the “correct” values (first/second row in the tables). For the constant image, projection method (i) (Table 1) showed a far too high Higuchi dimension 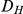 of approximately 1 instead of 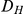 = 0. Extraction method (ii) (Table 2) led to a Higuchi dimension value estimation of 0.74. Almost identical values were gained for the cosine shaped image. The values for the predefined fractal images (*D* = 2.2, 2.5, 2.8) are drastically lowered, which is a clear consequence of the leading and tailing zeroes. Effectively, the values represent a mixture of both fractal dimensions (*D* = 2.2, 2.5, 2.8 and 0). Decreased values can also be seen for the random image. Overall, the influence of a rectangular ROI is very drastic and cannot be neglected. On the other hand, exclusion of the background (fourth row in the tables) compensated the ROI effects very well. The values for the non-fractal images are now nearly correct. Only the values for the fractal images and the random image are marginally higher.

The elliptical ROI caused distortions in case of including background (fifth row in the tables) compared to the “correct” values (first/second row in the tables), which are quite similar to the rectangular case. The details are not really of interest, because an arbitrary ROI would lead to an arbitrary background, influencing the results in an individual manner. More important is the question of whether it is possible to restore the values by eliminating the background. In contrast to a rectangular ROI, the background influence can not be eliminated in the same manner, especially for method (i) (sixth row in Table 1). A data point of the projection is only zero when and where all image pixels along the projection direction are zero. In fact, this condition holds only for pixels outside the surrounding rectangle of the ellipse. The areas inside the corners of the surrounding rectangle have zero values, and therefore the projection sums include these zero values, which evidently alter the determinations of the Higuchi dimension. Again, the elimination of the background resembled, with a high degree of conformity, the “correct” values.

Finally, using the extracted signals according to method (ii) (sixth row in Table 2), it was again possible to restore the values for the Higuchi dimension.

## Discussion

There are several accepted methods for determining the fractal properties of objects represented by digital images. The unavoidable drawback of digital images is the limited resolution. A pixel of an image is the smallest element, while the size of the image is the largest element of an image. Nevertheless, fractal analysis of digital images has been very successful in the past and can give reliable results with a high degree of validity [24], [25]. In contrary, this study showed that the Fourier method, which is commonly well suited for gray value images, performed rather poorly if solely regions of interests should be evaluated. The problem of the Fourier method is that it cannot be restricted to regions of interests at all. The discrete Fourier transformation of digital images is calculated with sums of all the elements in the individual rows and columns. A spatial data restriction is not compatible with discrete Fourier transformation.

In this study, an extension of the classical methods (e.g. Fourier dimension) for digital images has been proposed. This extension includes the use of fractal signal analysis and incorporates a time series evaluation method, developed for the determination and investigation of chaotic dynamical systems. The 2D digital images must be transformed into 1D signals, and the resulting gray level signal can be treated as if it were a time series signal.

The fractal dimensions of the 1D signals were calculated using the Higuchi method. Prior investigations included quite complex methods, such as phase space reconstructions. Especially, Mattfeld [20] proposed a method of stitching together 10 consecutive binary images of 510×510 pixels. The fraction of cells within 510 pixel long column perpendicular to the long axis gave the values for an 1D function. Despite the overall complexity, calculations were restricted to binary images. Contrary, calculations for the Higuchi method do not require a very high computational effort and can be implemented very fast for grey value images, without the need of image segmentation. Klonowski et al. [21] have already implemented the projection method according to (i) but comparisons to other extraction methods or the restriction to region of interests were not given.

In this study four 2D to 1D transformations have been thoroughly examined. The projection method (i) yields two 1D signals, which yield two values for the fractal dimension of one image: one for the x-axis and another for the y-axis. If the object in the digital image should be characterized by a single fractal dimension, an average of both values can be calculated. This average reflects the fractal dimension of the whole image, eliminating possible directional dependencies. For radially symmetric objects like fractal landscapes, both values are nearly identical. For other images, such as the image with a cosine shape in the x-direction and constant shape in y-direction, both values are different.

Therefore, the calculation of two directionally dependent fractal dimensions allows the distinguishing of directional dependencies, which cannot be resolved by classical 2D methods at all. In addition to this advantage, it is always possible to average the two different values and get a value identical to the classical methods.

The projection of the images according to method (i) naturally causes a reduction of information. Hence, only global characteristics of the object under investigation are examined. The actual values have been slightly lower than the real values. If fine details cannot be ignored, it is possible to avoid the projection by extracting every row and/or column and by calculating the corresponding means, according to method (ii). The computational effort is higher, but every individual value of every pixel is incorporated. Again, the method has the advantage of calculating directional dependencies, as well as the possibility of getting a single average value for the whole image. Orientation independent analyses can be carried out by using the extraction method (iii) or (iv). The calculation effort is lower than for method (ii), but the results are quite reliable. Particularly, the spiral extraction method gives a rotationally independent result without the need of calculating averages.

Moreover, the proposed methods can be applied to regions of interests only. By eliminating the leading and tailing zeros, it has been shown that the proposed 1D method estimates the fractal dimension very well. For arbitrary shapes of the regions of interests, it turned out, that the projection method according to method (i) should be avoided, because there is the possibility of summing up some zero values that are spatially located outside the ROI, but inside the surrounding rectangle. In these cases, it is necessary to use the extraction methods according to (ii) or (iii).

Despite the effectiveness of the proposed 1D extraction method, especially compared to the Fourier method, the limitation is obviously the indirect determination of fractal dimensions 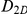
of two dimensional objects. In principle, for any one dimensional algorithm, 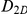 could be determined by adding 1 to 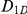,
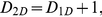
(9)but this may not be valid for every object, fractal or 2D to 1D extraction method. Considering practical aspects of recalculating 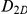 from 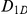, the influence of ROIs, especially for the case of projection, can be investigated by the following generalization:
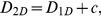
(10)
*c* being an experimentally derived constant. Since a ROI is a subset of the whole image, the fractal dimension of a ROI image (as far as discussed in this study) should be equal to the fractal dimension of the whole image. If at least one typical test image without any ROI is available, 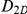 can be estimated with equation (9). If several typical test images are available (which is often the case), the mean could be calculated. Applying several typical ROIs on this test image or these test images yields *c*, by using equation (10). If *c* is known, the dimension 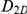 of a single image under investigation with a ROI can be calculated with equation (10).

### Conclusion

The fractal dimensions of objects in a digital image have been investigated by classical 2D methods, such as Box counting or Fourier methods, for a long time. Despite providing many reliable results, these methods have several restrictions, such as direction independence and the impossibility to restrict calculations to regions of interests. These shortcomings are especially problematic because the restriction to regions of interests is a very common task for biomedical images.

To overcome these limitations, this study proposes the transformation of 2D image data to 1D data series and the application of time series analyzing methods. The Higuchi dimension was calculated, and it has been possible to show that the proposed method is able to overcome the aforementioned shortcomings of classical 2D methods. It is possible to obtain directionally dependent fractal dimensions and, moreover, this approach can handle regions of interests very well.

The transformations to 1D signals have been carried out by four methods, but could be extended in future studies. Moreover, there is the great advantage that any conceivable 1D method, initially developed for time series analyses, can be adapted to investigate the spatial gray level information of digital images. In particular, it is intended to apply this method, as an example, to histological images of intraepithelial neoplasia, where a directional examination was not possible before. Prior quantitative examinations included the spatial shape and structure of nuclei [26], but it was not possible to consider their directional distribution throughout the epithelium. In addition, the possibility of restriction to regions of interests will decrease calculation errors and improve classification results. This method will certainly help the pathologist solve a long time diagnosis problem.
