# Correction: Higuchi Dimension of Digital Images

**DOI:** 10.1371/journal.pone.0119394

**Published:** 2015-04-10

**Authors:** The PLOS ONE Staff

In equation 4, the two brackets surrounding [N-m/d] should be replaced by floor function brackets. Please view the correct equation here.

$$L_{m}(d)=\frac{1}{d}\left\{\left(\sum_{i=1}^{\left\lfloor \frac{N-m}{d}\right\rfloor }\left|x(m+id)-x(m+(i-1)d)\right|\right)\frac{N-1}{\left\lfloor \frac{N-m}{d}\right\rfloor d}\right\},$$

In equation 5, the character “i” just under the summation sign should be replaced by “m”. Please view the correct equation here.

$$L(d)=\frac{1}{d}\sum_{m=1}^{d}L_{m}(d)$$

## References

[1] Ahammer H (2011) Higuchi Dimension of Digital Images. PLoS ONE 6(9): e24796 doi:10.1371/journal.pone.0024796 21931854 10.1371/journal.pone.0024796 PMC3172302

